# Nonsurgical Management of Perforating Internal Root Resorption in Maxillary Invaginated Lateral Incisor Using Cold Ceramic

**DOI:** 10.1002/ccr3.70526

**Published:** 2025-05-20

**Authors:** Zohreh Asgari, Neda Hajihassani

**Affiliations:** ^1^ Department of Endodontics, School of Dentistry Qazvin University of Medical Sciences Qazvin Iran

**Keywords:** bio‐ceramic materials, CBCT, dens invagination, nonsurgical, orthodontic treatment, perforating internal root resorption

## Abstract

The clastic activity results in internal root resorption (IRR), characterized by the progressive destruction of the surrounding dental hard tissues. This case reports perforating IRR in an advanced stage on a maxillary dens invaginated lateral incisor in a 13‐year‐old patient who completed orthodontic treatment 6 months ago. The perforating IRR was initially detected by periapical radiographs, and CBCT was used to accurately diagnose with full detail, enabling treatment planning and the identification of the extension and regions affected in the resorption lesion. A conservative internal approach was chosen for this case's management due to the periodontium's health and the absence of a periradicular lesion. After 1 year, the tooth was asymptomatic, and radiographic examinations confirmed no signs of periradicular pathology. Even for managing severe IRR cases, the internal approach can be a successful and minimally invasive treatment if providing an accurate procedure of treatment, the materials have good properties, and suitable equipment, such as bio‐ceramic materials and a dental operating microscope, are available.

**Summary**

- Successful perforated internal root resorption treatment utilizing a conservative internal approach with CBCT, a dental operating microscope, and bio‐ceramic materials.

## 1 Introduction

When clastic activity causes progressive destruction of intra‐radicular dentine and dentinal tubules along the middle and apical thirds of the canal walls, it is referred to as IRR [1]. Predentin and the outermost protective odontoblast layer are first disrupted in this pathologic process [2]. Only when a bacteriological stimulus—namely, the necrotic pulp tissue coronally to the defect and vital pulp tissue apically—is present in the canal does the inflammatory process continue [3].

The etiology and pathogenesis of this relatively uncommon pathology are still unclear [4]. However, several potential and likely risk factors have been suggested, including trauma, pulpitis, pulpotomy, a cracked tooth, tooth transplantation, restorative procedures, dens invagination, orthodontic treatment, and even a herpes zoster viral infection [5]. One of the most common developmental anomalies is dens invaginatus, which has a prevalence of between 0.3% and 10%. This anomaly sometimes leads to pulp necrosis, followed by IRR [6].

IRR is typically asymptomatic and discovered by chance during a radiographic examination [7]. The degree and stage of IRR determine its clinical characteristics. When the resorption progresses, the tooth is at least partially vital and can show typical pulpitis symptoms; however, most teeth with IRR are asymptomatic [8]. If the pulp becomes necrotic, untreated teeth can turn gray/dark gray [9]. A sinus tract typically develops due to the root perforation [8, 10]. IRR is indicated radiographically by a radiolucent, symmetrical, round enlargement of the root canal space, making the original canal shape indistinct [11, 12]. Conventional radiographic examination can only provide a two‐dimensional representation of a three‐dimensional object, which limits the diagnostic accuracy of IRR [13]. Cone‐beam computed tomography (CBCT) has been implemented in endodontics to improve the diagnosis and management of resorptive lesions [14].

IRR management is a challenge for endodontists, which, if left untreated, may destroy hard tissues adjacent to the tooth. When the root canal wall is perforated, the lesion can be sealed nonsurgically using the internal approach or with a combination of surgical access and orthograde root canal therapy. Materials such as MTA, Biodentin, and CEM can be used for this purpose [15]. Cold ceramic is a substance that resembles mineral trioxide aggregate (MTA) and has clinical uses comparable to those of other calcium silicate cements. In addition to being nontoxic and biocompatible, cold ceramic has an acceptable amount of radiopacity. It is used for a number of dental operations, including apical barrier formation in teeth with open apices, root‐end filling, root perforation repair, and potentially as a paste for abstracting root canals. In the presence of moisture, cold ceramic begins to set in 15 min and ultimately sets in 24 h [16, 17]. In addition to showing favorable cell attachment and high biocompatibility, cold ceramic also caused an increase in the expression of markers of osteo/odontogenic differentiation [18]. According to one study, cold ceramic has a more marginal adaptation than MTA for a long time [19].

This case report describes the successful nonsurgical treatment of a perforative IRR associated with a dens invaginated anterior tooth and a previous orthodontic treatment, representing the 1‐year outcome of treatment.

## 2 Case History/Examination

**FIGURE 1:**
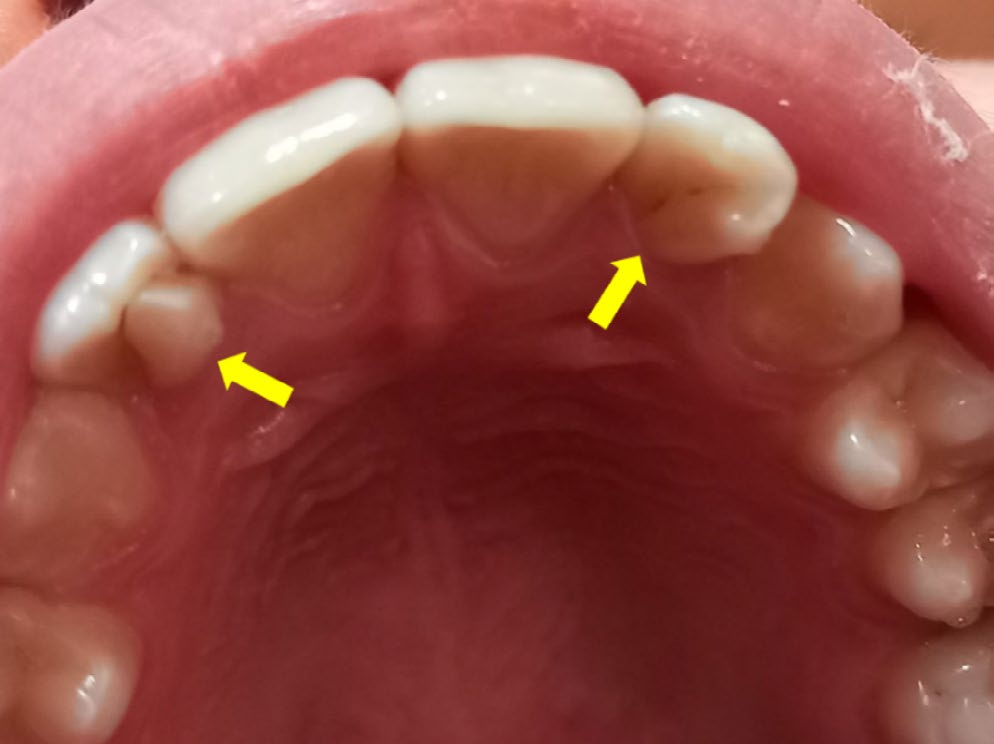
Both lateral incisors show a different palatal anatomy.

**FIGURE 2:**
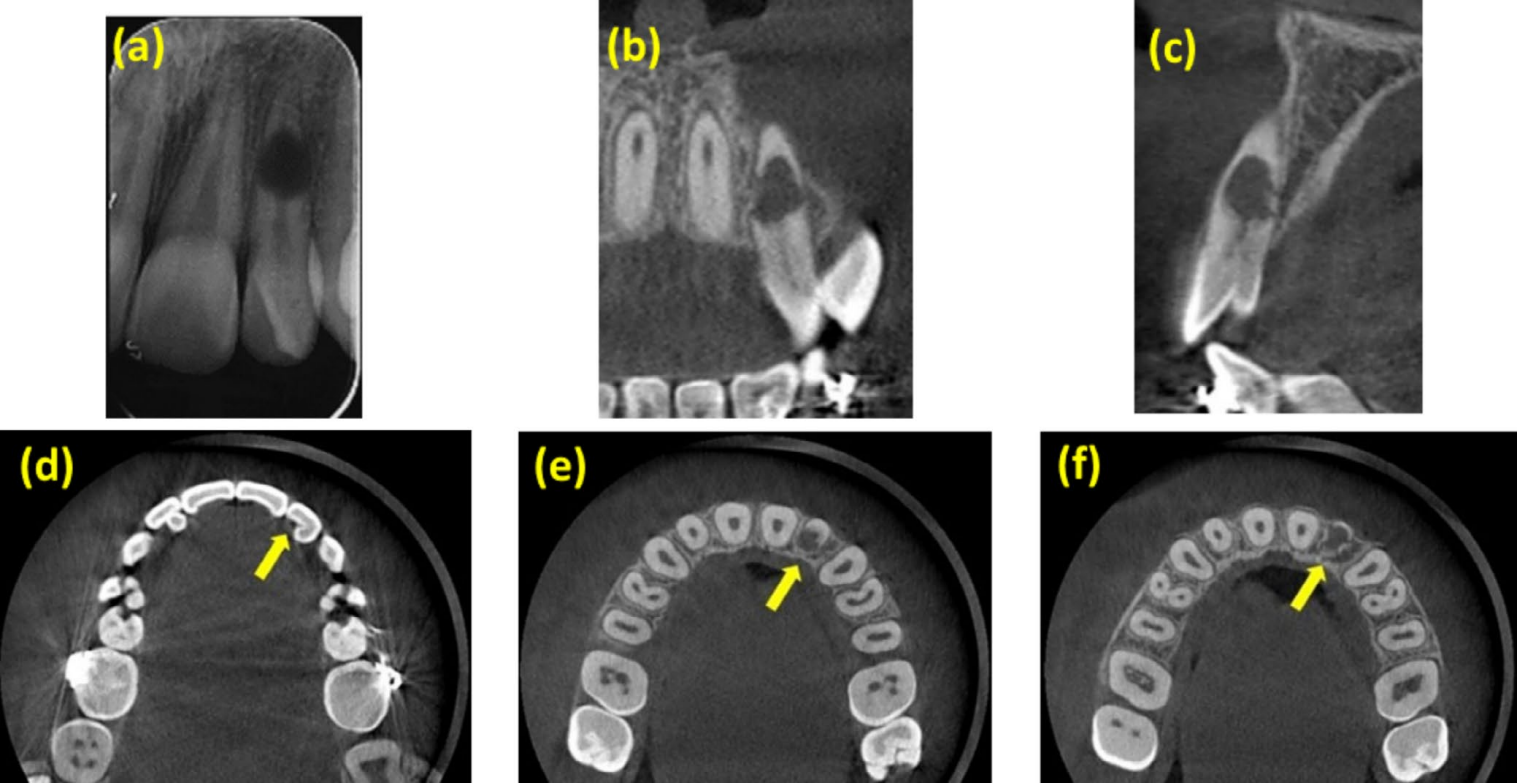
Periapical and CBCT image showing the extent of IRR in the maxillary left lateral incisor. (a) Preoperative periapical radiograph, (b) coronal view, (c) sagittal view, (d) dense invagination in axial view, and (e, f) root perforation in two different axial view.

A 13‐year‐old female patient was referred by an orthodontist to the endodontic clinic at the School of Dentistry, Qazvin University of Medical Science, chief complaining of recurrent pain and inflammation in the buccal region of the maxillary left lateral incisor for 1 month. The patient had no history of medical problems (ASA I). Intraoral examination revealed normal oral hygiene. Clinical examinations of this tooth discovered anatomical changes on the palatal surface known as dens invagination (Figure 1). Also, the contralateral tooth showed changes in the palatal surface anatomy (clinical examination and radiography of this tooth were normal). The patient had finished orthodontic treatment 6 months ago, and the lower incisors had a fixed ferrule metallic splint over the lingual surfaces. A large radiolucent lesion was discovered mid‐root of tooth 22 (maxillary left lateral incisor) by radiographic and tomographic examination, resulting in the loss of root continuity (Figure 2a). Moreover, the assessment of CBCT indicated the extent of the intracanal lesion (Figure 2b–f), which suggested that the inner pulpal space and the outer periradicular area communicated through multiple perforation zones. A large segment of the root structure was lost in the middle third of the root. The tooth mobility was within physiological ranges, with no periodontal pockets. The pulp vitality tests, including the heat (hot burnisher) and cold tests (Endo cold spray, Diamant, Iran), did not show any response from the tooth. There was no reaction to percussion, but the periradicular region was tender to palpation.

## 3 Diagnosis and Treatment

In addition to the radiographic diagnosis of perforating IRR, tooth 22 was clinically diagnosed with necrotic pulp and symptomatic apical periodontitis. The patient was informed about the clinical condition. After the suggestion of nonsurgical endodontic therapy, informed consent was obtained.

**FIGURE 3:**
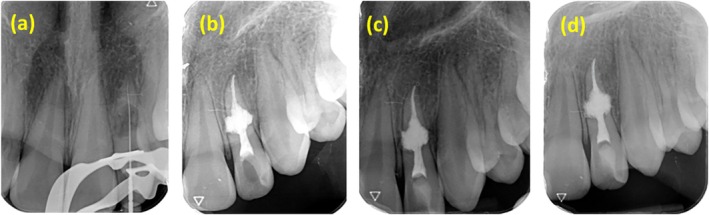
(a) Working length determination, (b) postoperative radiography with cold ceramic obturation, (c) 6‐month follow‐up, and (d) 12‐month follow‐up.

The tooth was anesthetized at the beginning of the treatment session using a buccal infiltration of 2% lidocaine containing 1:80,000 epinephrine. Then, a mouth was rinsed with 0.2% chlorhexidine gluconate, and a rubber dam was placed. Under a dental operating microscope (DOM) (Mediworks SM620, China), a classic access cavity was prepared utilizing a fissure diamond bur and a high‐speed handpiece. A # 15 K‐file (Mani Inc., Tochigi, Japan) was used to negotiate the canal, and granulation tissue with significant internal bleeding was observed. Working length was determined using an electronic apex locator (Root Zx II, Morita, USA) and confirmed by radiography (Figure 3a). The canal space was filled with 5.25% NaOCl using a 27‐gauge needle. Following, ProTaper Gold rotary file instruments (Dentsply, Sirona, USA) were used to prepare the canal space up to #F3 as the master apical file. The root canal granulation tissue was removed correctly, and the bleeding was controlled. In order to perform final irrigation, 1 mL of 17% EDTA (three 20‐s cycles) and then 5.25% NaOCl were ultrasonically activated. After that, the root canal was dried with sterile paper points. Calcium Hydroxide (EX Cidox, Nikdarman, Iran) powder was combined with normal saline to prepare a creamy paste, which was then applied to the canal using a lentulo spiral (Mani, Shioyagun, Japan) to dissolve the hyperemic resorptive tissue in the inaccessible areas. The access cavity of the tooth was restored with temporary cement.

On the patient's second visit (2 weeks following the first appointment), the temporary filling was removed after local anesthesia, and the tooth was isolated using a rubber dam. Under DOM magnification, the working length file and normal saline were employed to remove the calcium hydroxide paste from the root canal. Then, final irrigation was performed using 5.25% sodium hypochlorite with ultrasonic activation, followed by 1 mL of 17% EDTA, which was used intracanally for 1 min. The root canal was dried with sterile paper points. Using the ultrasonic activation of a plugger, the cold ceramic (Monsefteb, Yazd, Iran) was placed in the entire canal. In order to facilitate the proper setting of cold ceramic, moist cotton was placed in the canal orifice, and the temporary restoration was put in. Then, confirmation radiography was taken (Figure 3b).

Twenty‐four hours later, the cold ceramic setting was checked, and the tooth was restored using a composite resin adhesive; then, a scheduled follow‐up was adjusted for the patient.

## 4 Conclusion and Results

The patient had no symptoms after treatment, and radiographic examinations during the follow‐up indicated no signs of periradicular pathology. After 6 months (Figure 3c) and 12 months (Figure 3d), the patient reported no complications, and the tooth had normal function.

This case report illustrates the successful nonsurgical treatment of extensive perforating IRR using bio‐ceramic materials, especially cold ceramic. Clinical and radiographic follow‐up confirmed successful results with no symptoms and complete repair of the surrounding bone structure. In order to treat IRR, bioceramics have promising potential as substitutes for conventional materials.

## 5 Discussion

The present case demonstrated that a perforative IRR lesion in the upper left lateral incisor was successfully managed nonsurgically with cold ceramic. After 12 months, the tooth was symptom‐free and functional.

In the literatures, numerous factors, including orthodontics, trauma, caries, and heat, have been reported as potential causes of predentin layer damage and the potential outcome of IRR [20]. The etiology and pathogenesis of IRR are partially understood, and it is uncommon and hidden; however, it is known that for IRR to take place, the predentin and outermost protective odontoblastic layer must be damaged. This will expose the mineralized dentin underneath to odontoclasts [21]. There is scarce information regarding dens in dente that cause IRR [12, 22, 23]. Dens invagination or enamel layer defects may cause permeability or communication with the oral cavity, which damages the predentin layer and results in persistent pulp inflammation, ultimately leading to IRR [24].

According to studies, between 19% and 31.4% of patients receiving fixed appliance orthodontic treatment may experience orthodontically induced inflammatory root resorption [25]. Due to the orthodontic forces that compress the periodontal ligament (PDL), the most orthodontically induced root resorption affects the external root end surface, whereas intracanal changes cannot be ignored. The pulp is typically healthy in orthodontic‐related resorption, thoughthe resorption is unrelated to the pulpal conditions but rather associated with the tooth movement [4]. In this case, lateral tooth dens invagination and previous orthodontic movements are possible reasons that could cause the IRR.

The vitality of the pulp tissue apical to the resorption and partial or complete necrosis of the pulp tissue coronally are two essential and concurrent preconditions for IRR [3]. Although the necrotic tissue acts as a stimulant for the resorptive processes that the clastic cells mediate, the blood supply of the apical vital pulp supplies the clastic cells and nutrients. In the absence of this stimulus, resorption is a self‐limited mechanism [9].

If treatment is not received, necrosis develops throughout the canal, resulting in apical periodontitis. Most of the time, the teeth are symptom‐free, and routine radiographs reveal the defect. Making a differential diagnosis with external cervical resorption is necessary because this could influence the affected tooth's prognosis and treatment planning [20]. CBCT offers comprehensive and accurate information on the lesion's size, shape, and nature to enable appropriate planning, including any root perforations [26, 27]. One of the advantages of employing magnification is enhanced visualization through a microscope [28]. In this instance, it was helpful to visualize and precisely apply the material to the resorptive defect using the operating microscope during the treatment process.

When IRR is diagnosed, and the tooth is considered restorable with a reasonable prognosis, the preferred treatment is conventional root canal treatment [8, 29]. Obturation of the affected tooth is a significant clinical challenge; on the other hand, root canal therapy and access cavity preparation should be conservative to prevent further deterioration of the already compromised tooth. Furthermore, the resorption defect's shape challenges direct mechanical instrumentation [8, 10]. Another problem is that during the chemomechanical debridement, bleeding from the inflammatory granulation tissues may make it challenging to see vital teeth [29]. Due to calcium hydroxide being antibacterial and effective against treatment‐persistent bacteria, it is an excellent choice for this application. calcium hydroxide also functions synergistically with sodium hypochlorite to eliminate organic debris from the root canal [29, 30]. In this case, disinfection of the necrotic root canal was confirmed with calcium hydroxide as intracanal medication for 2 weeks.

In the next step, to avoid reinfection, a proper root‐filling material was used to obturate the canal. Because of intracanal irregularity, IRR defects can be challenging to obturate [31]. Therefore, a flowable obturation material is required, particularly when the root wall has been perforated [32]. Cold ceramic is nontoxic and biocompatible with host tissues, and it has good handling characteristics and consistency after mixing [16]. According to study outcomes, cold ceramic's sealing property is comparable to MTA in dry and saliva‐contaminated situations and superior to MTA in blood‐contaminated conditions. The setting time of cold ceramic is shorter than that of MTA, which was reported to be about 15 and 165 min, respectively. Additionally, there was no significant difference between MTA and cold ceramic regarding tooth discoloration [16].

A short follow‐up period was a limitation of our study, and we intend to report longer follow‐up results as they become available. The etiology and long‐term prognosis of this treatment plan require more investigation.

In addition to two‐dimensional radiography, CBCT is necessary for the preliminary assessment and diagnosis of resorption. However, CBCT is usually not required for continuous follow‐up; according to ALARA principles, intraoral periapical radiographs are appropriate [33].

## Author Contributions

**Zohreh Asgari:** conceptualization, data curation, investigation, methodology, resources, writing – original draft. **Neda Hajihassani:** project administration, resources, validation, visualization, writing – review and editing.

## Ethics Statement

This case report meets the ethical guidelines and adheres to Iran's local legal requirements.

## Consent

Written informed consent was obtained from the patient to publish this report in accordance with the journal's patient consent policy.

## Conflicts of Interest

The authors declare no conflicts of interest.

## Data Availability

The data that support the findings of this study are available on request from the corresponding author. The data are not publicly available due to privacy or ethical restrictions.
